# Three-dimensional aortic arch geometry and blood flow in neonates after surgical repair for aortic coarctation

**DOI:** 10.3389/fcvm.2024.1518070

**Published:** 2025-01-06

**Authors:** Katrin Fricke, Lea Christierson, Einar Heiberg, Pia Sjöberg, Erik Hedström, Kristoffer Steiner, Constance G. Weismann, Johannes Töger, Petru Liuba

**Affiliations:** ^1^ Pediatric Cardiology, Pediatric Heart Center, Skåne University Hospital, Lund, Sweden; ^2^ Pediatrics, Department of Clinical Sciences Lund, Lund University, Lund, Sweden; ^3^ Department of Biomedical Engineering, Lund University, Lund, Sweden; ^4^ Clinical Physiology, Department of Clinical Sciences Lund, Lund University, Lund, Sweden; ^5^ Department of Clinical Physiology, Skåne University Hospital, Lund, Sweden; ^6^ Diagnostic Radiology, Department of Clinical Sciences Lund, Lund University, Lund, Sweden; ^7^ Department of Radiology, Skåne University Hospital, Lund, Sweden; ^8^ Department of Women`s and Children`s Health, Karolinska Institute, Stockholm, Sweden; ^9^ Department of Pediatric Cardiology and Pediatric Intensive Care, Ludwig-Maximilian University, Munich, Germany

**Keywords:** neonatal coarctation, magnetic resonance, three-dimensional aortic arch geometry, four-dimensional flow, recurrent coarctation

## Abstract

**Background:**

Recurrent coarctation of the aorta (re-CoA) is a well-known although not fully understood complication after surgical repair, typically occurring in 10%–20% of cases within months after discharge.

**Objectives:**

To (1) characterize geometry of the aortic arch and blood flow from pre-discharge magnetic resonance imaging (MRI) in neonates after CoA repair; and (2) compare these measures between patients that developed re-CoA within 12 months after repair and patients who did not.

**Methods:**

Neonates needing CoA repair, without associated major congenital heart defects, were included. Transthoracic echocardiography (echo) and 4D phase-contrast MRI were performed prior to discharge after CoA repair to assess 3D arch geometry, flow velocity and flow pattern in the distal aortic arch corresponding to the area at risk for re-CoA. Arch geometry was assessed by measuring angles of the aortic arch and its branches using 3D patient-specific geometries segmented from MRI. Continuous data are presented as median and interquartile range.

**Results:**

The median age at CoA surgery was 9 days. Four out of the included 28 patients (14%) developed re-CoA within the first 12 months after surgery. Re-CoA was associated with repair technique (lateral thoracotomy 100% vs. 33%, *p* = 0.02), higher postoperative isthmic flow velocity by echocardiography (1.9 [0. 9] m/s vs. 1.25 [0.5] m/s, *p* = 0.04) and postoperative crenel aortic arch (100% vs. 21%, *p* = 0.007) with a larger distance between the first and last branching points (12.6 [3.1] mm vs. 7.3 [7.0] mm; *p* = 0.01). A smaller angle between the ascending aorta and the brachiocephalic artery (89 [58]° vs. 122 [37]°, *p* = 0.05) and between the proximal aortic arch and the left carotid artery (75° vs. 97 [37]°, *p* = 0.04), with a more pronounced caliber change between the ascending aorta and the proximal (1.85 vs. 0.86 [0.76]; *p* = 0.03) and distal aortic arch (2.19 [2.42] vs. 1.01 [0.94]; *p* = 0.03) were observed in re-CoA patients. Patients who developed re-CoA had more left-handed helical flow in systole (*p* = 0.045), more right-handed helical flow in diastole (*p* = 0.02), and less vortical flow (*p* = 0.05).

**Conclusion:**

Subtle changes in aortic arch geometry and flow pattern early after neonatal CoA repair may contribute to the risk of re-CoA.

## Introduction

Critical coarctation of the aorta (CoA) is a severe congenital heart defect (CHD) in need of immediate neonatal repair. If detected in time, the outcome after neonatal repair is usually good (1).

Recurrent aortic coarctation (re-CoA) is a well-known complication following neonatal CoA repair with an incidence of 10%–20% (2, 3). In most cases, re-intervention is needed within months after repair. The underlying mechanisms of re-CoA remain unclear. Previous studies have indicated a link between re-CoA and certain demographic, anatomic and surgical variables including younger age, and lower preoperative weight at repair, repair technique, smaller arch dimensions, increased postoperative peak Doppler gradient in the aortic isthmus and female sex (4–8). The shape of the aortic arch after CoA repair may also play a role; a previous study found an association between a pointed (gothic) or rectangular (crenel) aortic arch and an increased risk of re-CoA (9).

Magnetic resonance imaging (MRI) provides non-invasive assessment of aortic anatomy including angle measurements using 3D imaging. Flow patterns such as vortical and helical flow can be measured using four-dimensional (4D) phase-contrast MRI in healthy adults and in several groups at risk for aortopathy, including Marfan syndrome, CoA and relatives of patients with bicuspid aortic valve (BAV) (10–13). Sjöberg et al. showed that 4D flow MRI is feasible in neonates without the need for general anaesthesia or contrast agents (14). In addition, MRI-based three-dimensional (3D) volumes of the aortic arch allow a comprehensive imaging of the entire aortic arch and provide the basis for calculating 3D angles. This may be useful for modelling the complex aortic arch geometry in patients following neonatal CoA repair.

It may be hypothesized that postoperative changes in the aortic arch geometry may induce flow alterations in the distal aortic arch that could contribute to the formation of re-CoA.

Therefore, the aims were to (a) characterize aortic arch geometry and blood flow pattern in pre-discharge MRI in neonates after CoA repair; and (b) compare geometry and flow between those who did develop re-CoA early after repair and those who did not. In addition, we used a phantom setup to investigate the robustness of flow pattern analysis.

## Material and methods

This prospective study was conducted at the Children's Heart Center at Skåne University Hospital in Lund, one of two tertiary referral centers for pediatric heart surgery in Sweden. Neonates with CoA requiring surgical repair during the neonatal period were recruited between November 2018 and February 2023. Those with associated CHD other than ventricular septal defect (VSD) and BAV were excluded. Parental consent was obtained prior to study examinations. The Regional Ethical Review Board approved this study (2018/172). Pre- and post-operative clinical and echocardiography data, and post-operative MRI data, were acquired. Phantom data were acquired to investigate the robustness of the *in vivo* flow analysis.

### Preoperative examination

As part of the preoperative assessment, transthoracic echocardiograms (echo) were performed for diagnostic purposes and to assess associated intracardiac and aortic arch anomalies, while computed tomography was conducted at the surgeon's request to further delineate the aortic arch anatomy. The presence of BAV, VSD and any aortic arch anomalies were noted.

### Neonatal CoA-repair technique

Median sternotomy was performed in neonates with associated marked aortic arch hypoplasia and/or the concomitant presence of a moderate to large VSD requiring surgical treatment. In the remaining cases, lateral thoracotomy was performed. All patients underwent echo and MRI after CoA repair prior to discharge.

### Postoperative echocardiography

Echo (Epiq 7, Philips Medical System, Andover, USA) and cardiac MRI were usually performed on the same day. Flow velocities in the aortic isthmus, measured by continuous Doppler, were recorded.

### Postoperative MRI

Cardiac MRI was performed using the “feed-and-sleep” technique and sedation with rectally administered chloral hydrate (25 mg/kg, APL, Sweden) as by clinical routine when necessary (14, 15). Examinations were performed on a 1.5 T MAGNETOM Aera scanner (Siemens Healthineers, Forchheim, Germany). A T1-weighted black blood 3D sequence for vessel anatomy and a 4D phase-contrast flow research sequence were acquired. Both included the aorta from below the valve plane to diaphragm level. For the 3D black blood sequences, the acquired and reconstructed spatial resolution was 0.85 × 0.85 × 0.5 mm^3^, respectively. The 4D flow had an acquired spatial resolution of 2.4 × 3.6 × 1.5 mm^3^ and reconstructed spatial resolution of 2.4 × 2.4 × 1 mm^3^. The acquired temporal resolution was 42 ms or 9–13 frames per heartbeat and reconstructed spatial resolution 20 frames per heartbeat. The temporal segmentation factor was 2 and repetition time 5.2 ms, echo time 2.5 ms and flip angle 7°. The velocity encoding parameter was 150 cm/s, and acceleration method GRAPPA with *R* = 2 was used. Full 4D flow sequence parameters are shown in Supplementary Table S1.

Cardiac cycle gating in patients was done using ECG and in the phantom by connecting a digitally activated red light emitting diode (LED) to the scanner peripheral pulse unit (PPU). Phase background correction was applied in all 4D flow datasets, whereas phase unwrapping was not needed. Detailed black blood and 4D flow sequence parameters can be found in our previous study (14). Contrast agent was not used. A research version of Segment v3.3 R12067 (Medviso AB, Lund, Sweden) with custom plug-ins for 4D flow analysis was used for image analyses (15). All data were anonymized and observers (EiH, LC, KF) were blinded to the postoperative clinical outcome of the patients.

#### Assessment of aortic arch geometry

Three-dimensional (3D) models of the aortic arch were segmented from black blood images using Segment 3DPrint software v4.0 R12472 (Medviso AB, Lund, Sweden). The images were first resampled to an isotropic resolution of 0.5 × 0.5 × 0.5 mm^3^, from which the 3D aortic models were semi-automatically segmented by local thresholding by an observer (EiH, 20 years of experience in cardiac image segmentation). The aorta was then dilated by one pixel and smoothed with a 0.9 mm Gaussian kernel. The dilation was performed to counteract the effect of diameter shrinkage in the smoothing process. The smoothing was performed to facilitate three-dimensional angle measurements. As a final quality control, the models were evaluated in all slices in all three planes (transverse, sagittal and coronal) and in 3D. If necessary, corrections were made to the non-dilated and unsmoothed version and the dilation and smoothing process was repeated. The anatomical models were exported in STL format and further processed for angle measurements in the Vascular Modelling Toolkit (VMTK).

##### 1. Aortic arch shape

**Figure 1:**
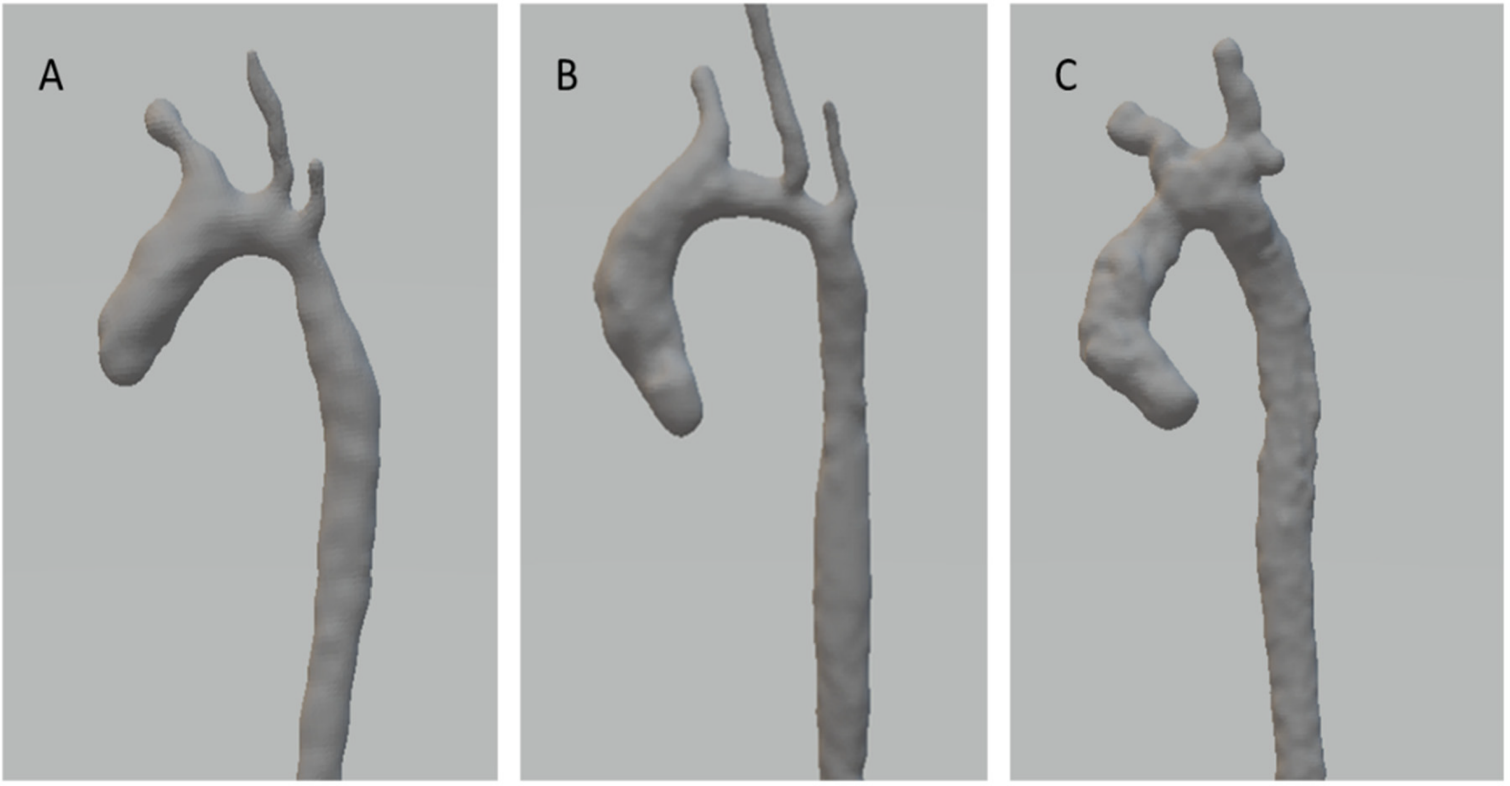
Subtypes of postoperative aortic arch shape. **(A)** Roman, **(B)** Crenel, **(C)** Gothic.

The aortic arch shape of the 3D models was qualitatively categorised by a single observer (KF) as (A) roman (round), (B) crenel (rectangular appearance and a long transverse arch segment), or (C) gothic (acute angle and short distance between the ascending and descending aorta) (Figure 1) (16).

##### 2. 3d angles of the aortic arch

**Figure 2:**
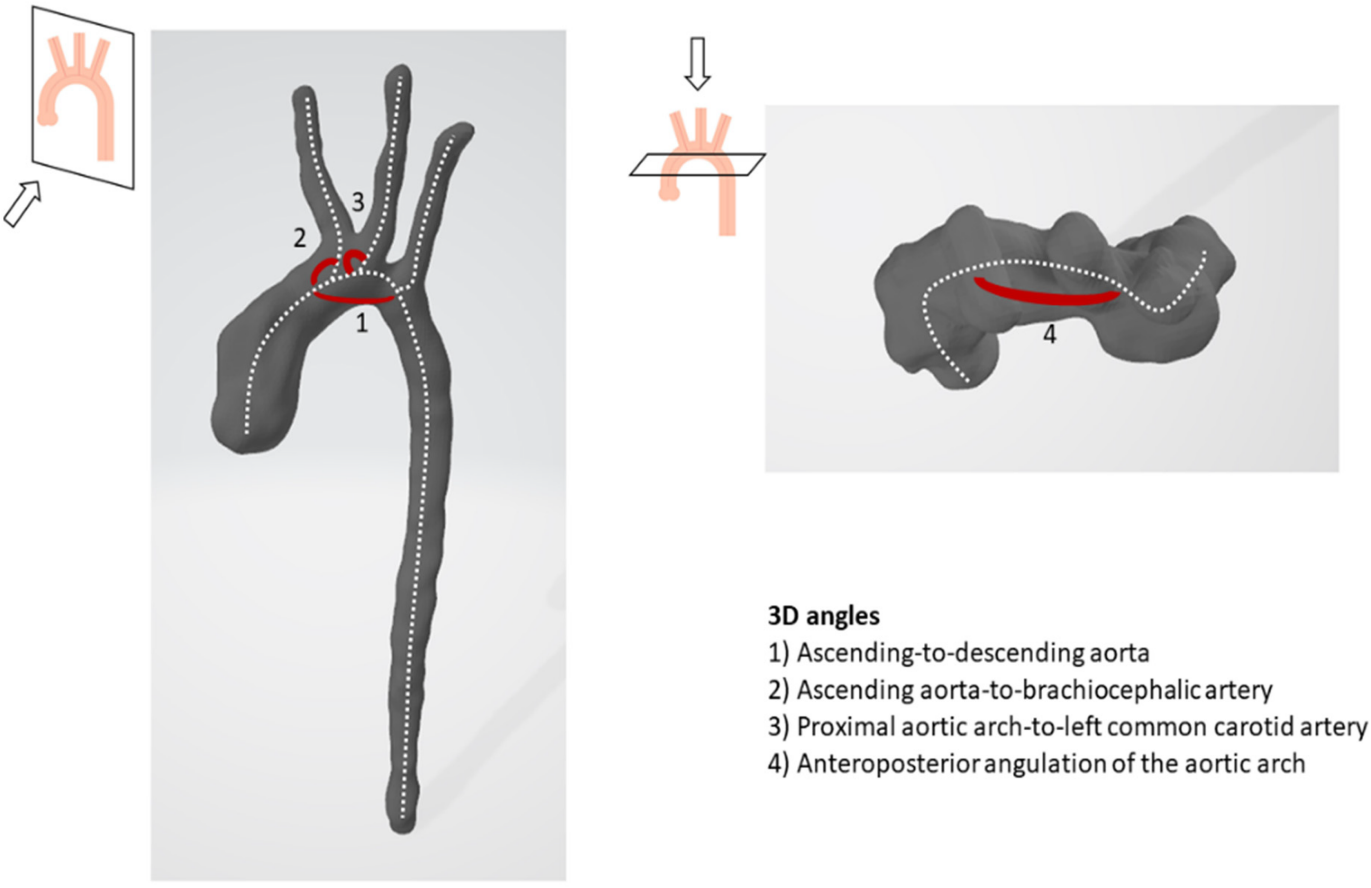
Illustration of 3D angles of the aortic arch.

The 3D angles of the aortic arch were computed based on the 3D models using the centerline of the geometry. At points of bifurcation, vectors were computed based on the tangent of the centerline of each bifurcating branch which were used to determine the bifurcation angle of each branch. Using the centerline, the angle of the aortic arch and its anterior-posterior angulation were determined, as well as the distance between the first and third bifurcations. Figure 2 visualizes measured 3D angles. All measurement were conducted by a single observer (LC).

##### 3. Caliber changes from the distal ascending aorta to the aortic isthmus

The aortic areas were measured proximal to the origins of the brachiocephalic artery (corresponding to the distal segment of the ascending aorta), left common carotid and left subclavian artery, and at the level of the aortic isthmus. The relative change in caliber was measured as a ratio of the distal ascending aorta area to the aortic area at these three levels.

#### Assessment of flow pattern in the distal aortic arch

**Figure 3:**
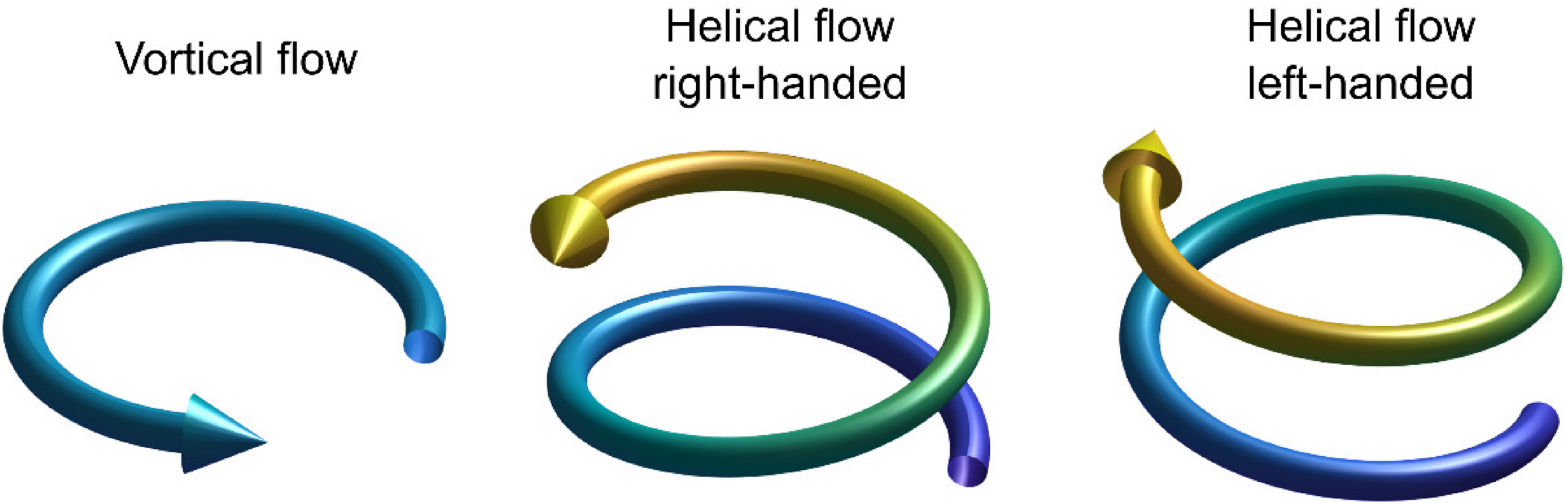
Schematic illustration of vortical flow, right-handed helical flow, and left-handed helical flow.

Vortical flow was defined as a rotational movement of the blood around a center point, while helical flow is a corkscrew-like rotation of the blood along the direction of blood flow in the vessel. Helical flow can be right-handed or left-handed (Figure 3). Quantitative vortical and helical flow measurements were made right after the left common carotid artery bifurcation to 2 cm distal to the left subclavian artery bifurcation. When a bovine arch was present, measurements were carried out distal to the innominate artery bifurcation.

Quantitative vortical and helical flow measurements were made using an in-house plugin to the Segment software, implementing the vector pattern matching method proposed by Heiberg et al. (17). In short, this method is used to determine how well the flow field matches patterns with idealized vortex cores and helical flow, respectively. The similarity is measured on a scale of 0–1, where 0 is no match and 1 is the local flow field perfectly matching the patterns.

The 4D flow data was resampled to 1 mm isotropic resolution, and the vortex and helix detection was performed with a filter radius of 2.75 mm and a filter roll-off factor *σ* = 1,000. Helical and vortical flow measurements are displayed as average over a cardiac cycle, in systole (average), diastole (average), and peak values. All measurements were performed by a single observer (KF).

#### Resolution sensitivity of vortex analysis in flow phantom

To investigate the sensitivity of the vortex analysis to 4D flow data spatial resolution, a 3D-printed phantom model of the neonatal aortic arch of one of the patients was constructed from MR images as previously described by Sjöberg et al. (14). Pulsatile flow at 120 beats per minute was applied using an in-house custom-built pump, also described in more detail previously (14). Flow data was acquired in 3 different resolutions (1 × 1 × 1 mm^3^, 2 × 2 × 2 mm^3^, and the clinically used sequence with 2.4 × 3.6 × 1.5 mm^3^ voxels, respectively) and all resampled to 1 × 1 × 1 mm^3^. Sequence parameters are provided in Supplementary Table S1. Helical and vortical flows were calculated and compared. All measurements were performed by a single observer (JT). Differences between helical or vortical flow curves for different spatial resolutions were quantified by taking the mean difference over all time frames in the flow data, divided by the mean value for the clinically used sequence, and expressed in percent.

### Outcome

Re-CoA was defined as a narrowing of the aortic arch requiring re-intervention within the first 12 months after CoA repair, routinely performed via balloon angioplasty.

### Statistics

Continuous data are presented as median and inter-quartile range [IQR]. The Mann–Whitney-*U*-test was applied for groups-wise data comparison. For categorical variables, the *χ*^2^ test or, in case of a small sample size, the Fisher's Exact test was applied. For linear relation, Pearson's correlation was calculated. *P*-values ≤0.05 were considered to indicate statistically significant differences. All analyses were performed using the Statistical Package for Social Sciences, version 29 (IBM SPSS, Chicago, IL).

## Results

In total, 36 neonates were included. Sedation was needed in (18/36) 50% of patients, whereas feeding alone was sufficient in all other patients. Of these, 7 were excluded due to suboptimal MRI data due to motion and 1 was excluded due to associated critical aortic stenosis. Hence, complete MRI and echo data were available in 28 neonates.

Patient characteristics are presented in Table 1. The median gestational age and weight at birth were 39.6 [2.2] weeks and 3,270 [739] grams. Associated VSDs and BAV were present in 14 (50%) and 19 (68%) patients, respectively. Pronounced preoperative aortic arch hypoplasia was noted in 20 patients (71%) and bovine aortic arch in 4 patients.

**Table 1:** Patient characteristics.

Variables	Median [IQR] or *n* (%)
Postnatal data	
Gestational age at birth (weeks)	39.6 [2.2]
Weight at birth (grams)	3,270 [739]
Female sex (%)	14 (50)
Associated cardiac anomalies	
VSD (%)	14 (50)
BAV (%)	19 (68)
Aortic arch hypoplasia (%)	20 (71)
Bovine arch (%)	4 (14)
CoA repair	
Age at repair (days)	9 [11]
Lateral thoracotomy (%)	12 (43)
Type of repair (%)	
End-to-end	4 (14)
Extended end-to-end	7 (25)
End-to-side	6 (21)
Arch repair with homograft patch	11 (39)
Postoperative	
Age at MRI (days)	16 [9]
Weight at MRI (grams)	3,410 [990]
Weight <2.5 kg at MRI (%)	2 (8)
Isthmic flow velocity by echo (m/s)	1.4 [0.6]
Systolic blood pressure gradient arm/leg (mmHg)	−3 [15]*
Re-CoA within 12 months	4 (14)
Age at diagnosis of re-CoA (days)	3.5 [1.2]

Data are presented as median [IQR] or *n*/*N* (%).

*n*, number of patients for given variable; *N*, total number of patients; *n* = 28; *, *n* = 27; BAV, bicuspid aortic valve; re-CoA, recurrent coarctation; VSD, ventricular septal defect.

### Surgical data

Lateral thoracotomy was carried out in 12 (43%) patients, including 4 patients with end-to-end, 7 with extended end-to-end and 1 patient with end-to-side repair. Median sternotomy was deemed necessary in the remaining 16 (57%) patients, including 5 with end-to-side anastomosis and 11 patients with aortic arch repair using homograft patch material (Table 1). Eight patients underwent concomitant VSD closure, and one had an additional VSD surgery at 2 months of age.

### MRI assessment

MRI was conducted at a median age of 16 [9] days and a weight of 3,410 [990] grams at a median of 6.5 [3] days after neonatal CoA repair. Of the included 28 patients, 17 had a roman, 9 a crenel and 2 a gothic aortic arch shape. The type of neonatal CoA repair was not associated with the postoperative arch shape (*p* = 0.2, data not shown).

#### Relation between aortic arch shape, 3d angles and flow pattern

Patients with crenel arch had a larger angle between the ascending and descending aorta (117 [13.9]° vs. roman 109 [10.8]° vs. gothic arch 97°; *p* = 0.018), a larger distance between the first and last branching points (11.9 [3.6] mm vs. roman 7.1 [4.7] mm vs. gothic 3.4 mm; *p* = 0.003) and a larger angle between the distal aortic arch and the left subclavian artery (120.5 [22.0]° vs. roman 102.6 [43.5]° vs. gothic 66.2°; *p* = 0.03) compared to the other 2 shapes. Patients with gothic arch had more peak vortical flow in the distal aortic arch than those with other aortic arch shapes (gothic 0.193 vs. roman 0.167 [0.045] vs. crenel 0.138 [0.029]; *p* = 0.03). No difference in helical flow was found between the three groups.

**Figure 4:**
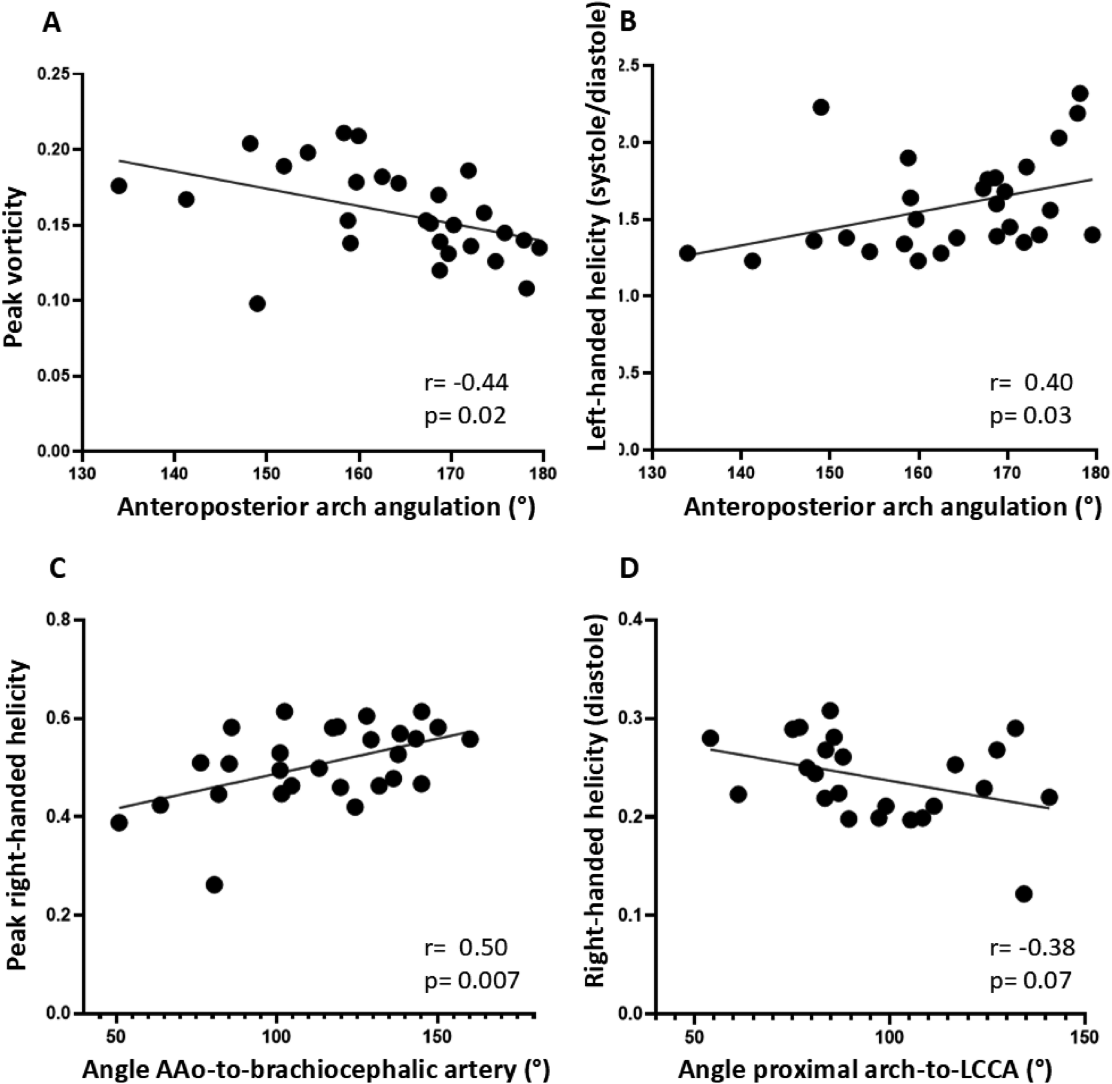
Relationship between postoperative aortic arch geometry and flow pattern. **(A)** and **(B)**: Relationship between anteroposterior arch angulation and flow pattern in the distal aortic arch to **(A)** peak vortical and **(B)** left-handed helical flow. **(C**) Relationship between the angle between the ascending aorta (AAo) and the brachiocephalic artery to peak right-handed helical flow. **(D)** Relationship between the angle between the proximal aortic arch and the left common carotid artery (LCCA) and right-handed diastolic flow.

As the angle between the ascending and descending aorta increased, there was a trend towards more left-handed helical flow in diastole (Pearson's correlation coefficient 0.362; *p* = 0.056). With increasing anteroposterior arch angulation, more peak and diastolic vortical flow and less left-handed helical flow was observed (Figures 4A,B). As the angle between the ascending aorta and the brachiocephalic artery increased, peak right-handed helical flow increased, and as the angle between the proximal aortic arch and the left common carotid artery increased, diastolic right-handed helical flow decreased (Figures 4C,D).

#### Relation between caliber change and flow pattern

The postoperative change in caliber between the ascending aorta just before the brachiocephalic artery branching point and the distal aortic arch just before the left subclavian artery branching point was negatively associated with vortical flow (peak: Pearsońs correlation coefficient −0.35; *p* = 0.015; diastolic: Pearson’s correlation coefficient −0.39; *p* = 0.04) and with right-handed helical flow (peak: Pearsońs correlation coefficient −0.46, *p* = 0.01; systolic: Pearson's correlation coefficient −0.38, *p* = 0.04).

#### Recurrent coarctation (re-CoA)

Re-CoA occurred in 4 patients at a median age of 3.5 [1.2] months (Table 1). The main characteristics of patients with and without re-CoA are summarised in Table 2. There were no differences between these 2 groups in gestational age, birth weight, sex, age at repair, presence of associated cardiac anomalies (VSD and BAV) and bovine aortic arch.

**Table 2:** Characteristics of patients with and without early re-CoA.

Variables	No re-CoA (*n* = 24) [IQR] or *n* (%)	re-CoA (*n* = 4) [IQR] or *n* (%)	*p*
Postnatal data			
Gestational age at birth (weeks)	39.6 [2.2]	39.3 [3.5]	0.7
Weight at birth (grams)	3,337 [681]	2,850 [953]	0.12
Female sex (%)	12 (50)	2 (50)	0.7
Associated cardiac anomalies			
BAV (%)	18 (75)	1 (25)	0.08
VSD (%)	12 (50)	2 (50)	1
Preoperative arch hypoplasia (%)	19 (79)	1 (25)	0.058
Bovine arch (%)	3 (13)	1 (25)	0.5
CoA repair			
Age at repair (days)	9 [11]	5.5 [6]	0.12
Lateral thoracotomy (%)	8 (33)	4 (100)	0.02
Type of repair (%)			
End-to-end	2 (8)	2 (50)	0.04
Extended end-to-end	5 (21)	2 (50)	
End-to side	6 (25)	0 (0)	
Aortic arch repair	11 (46)	0 (0)	
Associated VSD-operation (%)	8 (33)	0 (0)	0.3
MRI and echo assessment			
Age at assessment (days)	17.5 [10]	10.5 [5]	0.008
Gestational age (weeks)	42.3 [1.9]	40.6 [2.6]	0.04
Weight (grams)	3,400 [811]	3,125 [1,565]	0.4
Weight <2,500 grams (%)	1 (4)	1 (25)	0.3

MRI-derived variables	No re-CoA (*n* = 24) [IQR] or *n* (%)	re-CoA (*n* = 4) [IQR] or *n* (%)	*p*
(A) Aortic arch geometry			
Postoperative arch shape (%)			
Roman	17 (71)	0 (0)	0.007
Gothic	2 (8)	0 (0)	
Crenel	5 (21)	4 (100)	
3D angles (°) and distances (mm)			
AAo-to-DAo	110.8 [13.3]	119.4 [15.3]	0.15
Anteroposterior arch angle	165.8 [16.6]	168.8 [12.0]	0.6
AAo-to-brachiocephalic artery	121.7 [37.1]	88.6 [57.9]	0.05
Proximal arch-to-LCCA	97.2 [37.0]	75.1 [−]	0.04
Distal arch-to-LSA	103.3 [46.4]	116.4 [23.7]	0.1
Distance first-to-last branching points	7.31 [6.98]	12.56 [3.06]	0.01
Arch angle-to-distance	15.11 [15.88]	9.06 [2.24]	0.02
Caliber change			
AAo-to-proximal aortic arch	0.86 [0.76]	1.85 [−]	0.03
AAo-to-distal aortic arch	1.01 [0.94]	2.19 [2.42]	0.03
AAo-to-aortic isthmus	1.50 [0.89]	1.74 [1.53]	0.5
(B) Helical & vortical flow			
*Peak* right-handed helical flow	0.518 [0.119]	0.485 [0.119]	0.3
Mean right-handed helical flow	0.343 [0.048]	0.356 [0.071]	0.4
Right-handed helical flow in systole	0.453 [0.095]	0.441 [0.095]	0.8
Right-handed helical flow in diastole	0.227 [0.061]	0.281 [0.015]	0.018
Right-handed helical flow (S-to-D)	1.993 [0.56]	1.548 [0.41]	0.018
*Peak* left-handed helical flow	0.385 [0.061]	0.434 [0.059]	0.12
Mean left-handed helical flow	0.269 [0.080]	0.313 [0.052]	0.06
Left-handed helical flow in systole	0.337 [0.101]	0.394 [0.060]	0.045
Left-handed helical flow in diastole	0.212 [0.070]	0.251 [0.051]	0.1
*Peak* vortical flow	0.162 [0.047]	0.133 [0.028]	0.07
Mean vortical flow	0.117 [0.026]	0.098 [0.022]	0.07
Vortical flow in systole	0.103 [0.028]	0.089 [0.027]	0.16
Vortical flow in diastole	0.127 [0.025]	0.106 [0.020]	0.05
Isthmic flow velocity prior to discharge (transthoracic echo) (m/s)	1.25 [0.50]	1.9 [0.89]	0.035
Systolic blood pressure gradient arm-to-leg prior to discharge (mmHg)	−3.0 [10]	1 [21]	0.2

Data are presented as median [IQR], *n/N* (%).

*n,* number of patients for given variable; *N*, total number of patients; AAo, ascending aorta; DAo, descending aorta; LCCA, left common carotid artery; LSA, left subclavian artery; S-to-D, systole-to-diastole; BAV, bicuspid aortic valve; re-CoA, recurrent coarctation; VSD, ventricular septal defect; No re-CoA: *n* = 24 (except for blood pressure gradient: *n* = 23); re-CoA, *n* = 4 (except for angle between the proximal arch and brachiocephalic artery *n* = 3).

#### Surgical technique and re-CoA

All patients with re-CoA had repair via lateral thoracotomy, either with end-to-end or with extended end-to-end anastomosis (Table 2). Neither young age (<15 days) nor low preoperative weight (<2.5 kg) at the time of CoA repair were associated with re-CoA (data not shown).

#### Aortic arch geometry and flow pattern in relation to re-CoA

**Figure 5:**
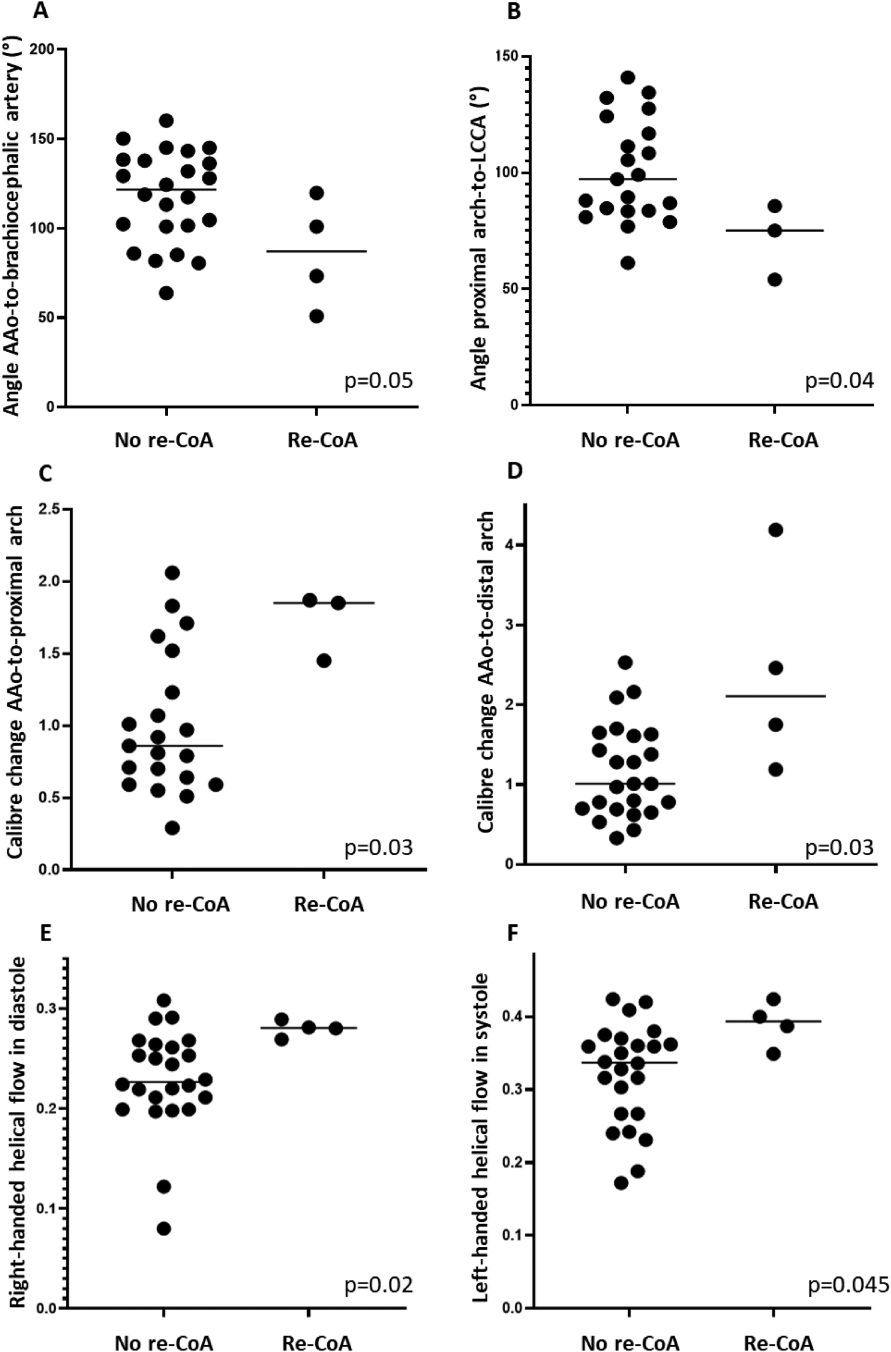
Variables in relation to outcome re-CoA. **(A)** Angle between the ascending aorta (AAo) and brachiocephalic artery. **(B)** Angle between the proximal aortic arch and left common carotid artery (LCCA). **(C)** AAo-to-proximal aortic arch caliber change **(D)** AAo-to-distal aortic arch. **(E)** Right-handed helical flow in diastole. **(F)** Left-handed helical flow in systole.

All patients with re-CoA had a crenel arch after neonatal CoA repair (Table 2). In addition, a smaller angle between the ascending aorta and the brachiocephalic artery and between the proximal aortic arch and the left common carotid artery was found in re-CoA patients (Table 2, Figures 5A,B). The change in aortic arch caliber was greater in patients with re-CoA than in those who did not develop re-CoA (Table 2, Figures 5C,D). More left-handed systolic helical flow, more right-handed diastolic helical flow, and less vortical flow were noted in patients with re-CoA (Table 2, Figures 5E,F).

#### Pre-discharge blood pressure, echo data and re-CoA

Data are shown in Table 2. Prior to discharge after CoA repair, there was no significant difference in the systolic blood pressure gradient between arms and legs. Isthmic flow velocity measured by echo was higher in patients with re-CoA (Table 2).

#### Resolution sensitivity of vortex analysis in flow phantom

A visualization of vortical and helical flow for different spatial resolutions is shown in Supplementary Figure S1. Visually, vortical flow showed comparable results between the three spatial resolutions (1 × 1 × 1 mm^3^, 2 × 2 × 2 mm^3^, patient protocol: 3.6 × 2.4 × 1.5 mm^3^). Left-handed and right-handed helical flow appears visually to have lower values for 1 × 1 × 1 mm^3^ compared to 2 × 2 × 2 mm^3^ and the patient protocol. Supplementary Figure S2 shows quantification of the vortical and helical flow over the phantom pump cycle is shown in. Compared to the patient protocol, the 2 × 2 × 2 mm^3^ protocol showed a difference of −4% for vortical flow, −2% for right-handed helical flow and −7% for left-handed helical flow. For the 1 × 1 × 1 mm^3^, the differences were −37%, −60% and −59%, respectively.

## Discussion

There is no previous study combining geometry and flow patterns in the aortic arch early after neonatal CoA repair. As current routine screening methods are unable to predict the risk for re-CoA after neonatal CoA repair, the current study adds knowledge by indicating an association between aortic arch geometry and flow pattern in the distal aortic arch following neonatal CoA repair. Although the number of infants who developed re-CoA was small (*n* = 4), there were several differences in 3D arch geometry and flow compared to infants without re-CoA.

### Aortic arch geometry and flow patterns

Three different subtypes of aortic arch shape following CoA repair have been described in the literature: a round (roman), a rectangular (crenel), and a pointed arch with an acute angle between the ascending and descending aorta (gothic) (16). Previous studies have linked the gothic arch to higher risk for systemic hypertension, increased left ventricular (LV) mass and decreased LV function compared to other aortic arch configurations (18–21). Earlier studies have focused on the angles between different segments of the aortic arch or between the distal ascending aorta and the proximal descending aorta (9, 16, 22). However, a more complex model of the aortic arch morphology may be required to assess its influence on haemodynamics (23). In this study, T1-weighted black blood MRI sequences were used to reconstruct the 3D geometry of the aortic arch, which enables precise anatomic delineation and calculation of angles in multiple planes (Figure 1). This may provide a more precise assessment of aortic arch geometry in comparison to earlier studies.

The use of 4D flow may aid to visualize changes in the aortic arch flow pattern, including vortical and helical flow (10–13). The application of 4D flow in the aortic arch is feasible even in neonates, as demonstrated in a recent study from our center (14). In contrast to previous studies (11–13, 24), we quantitatively assessed flow patterns using the 3D vector matching method proposed by Heiberg et al. (17). The use of quantitative measurement methods may lead to more reliable and comparable results regarding vortical and helical flows.

Comparing the quantitative vortical flow patterns between different resolutions in the flow phantom, the patient protocol (3.6 × 2.4 × 1.5 mm^3^, mean 2.5 mm) agreed with the 2 × 2 × 2 mm^3^ protocol. This shows that quantification of vortical and helical flow patterns is repeatable and robust with respect to variations in the acquisition protocol. This is important as previous 4D flow studies have shown significant differences in quantitative results between different 4D flow protocols (25). In contrast, the 1 × 1 × 1 mm^3^ resolution shows lower quantitative vortex and helix values compared to the 2 × 2 × 2 mm^3^ resolution and the patient protocol. This can be explained by the fact that the lower resolution leads to a smoothing of the vortical flow values to a larger proportion of the aortic lumen and therefore the mean values are higher for the lower resolutions. Although scanning at 1 × 1 × 1 mm^3^ resolution *in vivo* is not feasible with current 4D flow methods, advances in 4D flow imaging and reconstruction enabling higher resolution may lead to a more detailed and accurate view of blood flow patterns in neonates. Therefore, we suggest using protocols with approximately 2 mm voxel size with currently available technology.

In healthy adults with a left-sided aortic arch, dominant right-handed systolic helical arch flow and right-handed diastolic helical flow in the descending aorta are common (26). Frydrychowicz et al. noted helical aortic arch flow, predominantly right-handed, in approximately 60% of healthy adults, especially when the arch had normal configuration (roman shape with crook shape) (13). A vortical flow pattern in the aortic arch was found in only 10% of healthy grown-ups. With age, a decrease in helical flow or even a change to left-handed helical flow and an increase in vortical flow were observed, probably due to a loss of aortic wall elasticity (13). In agreement with these two studies, right-handed helical flow was the dominant flow pattern in the distal aortic arch whereas vortical flow was rare in our cohort.

### Relationship between postoperative aortic arch geometry and flow pattern

#### Aortic arch shape, 3d angles and flow pattern

Infants with a crenel aortic arch configuration exhibited larger angles between the ascending and descending aorta and a greater distance between the first and last aortic arch branching points. Despite the pronounced peak vortical flow in infants with gothic arch, no significant difference in flow pattern was found between the three groups. This may change with age, as less right-handed helical flow and more vortical flow appear to be predominate in healthy adults with a gothic or crenel arch (13). Similar to our study, Quail et al. found an association between subjective identification of a gothic arch and arch angulation, but not haemodynamics, in adults following CoA repair in the first year of life (23). This may suggest that qualitatively assessed arch shape has little influence on the flow pattern in the distal aortic arch.

Increasing anteroposterior arch angulation was associated with decreasing vortical and increasing left-handed helical flow in our study. To the best of our knowledge, these findings have not been described before. One could speculate that with increasing anteroposterior arch angulation, changes in flow pattern in the distal aortic arch, with potential clinical implications including re-CoA, could occur. This needs to be confirmed in future studies.

Another finding, not reported before, was the association between a greater angle between the ascending aorta and the brachiocephalic artery with more peak right-handed helical flow. Again, understanding the implication of this finding in clinical setting requires further work.

#### Caliber and flow pattern changes

A smaller distal arch compared to the distal ascending aorta was associated with less peak and diastolic vortical flow and with less peak and systolic right-handed helical flow in the distal aortic arch. This may suggest that a wider distal arch, typically found after arch reconstruction with patch, allows for more vortical and helical flow. Right-handed helical flow is the predominant flow pattern in the aortic arch in healthy adults (26) and we suspect that it is less common in patients with caliber mismatch and a smaller arch following neonatal CoA repair, as helical flow may not be able to continue undisturbed in the narrow aortic arch. Consistent with our findings, Quail et al. found an association of larger ascending aorta and smaller aortic arch and descending aorta with flow anomalies (23).

### Variables in relation to outcome of re-CoA

Four out of 28 patients (14%) needed reintervention due to re-CoA, which is comparable to other studies (2, 3, 27, 28).

#### Associated minor CHD and their association with re-CoA

The coexistence of minor CHD as BAV or VSD was not predictive for re-CoA, which is in line with earlier studies (4–6). In contrast to prior studies (29), a bovine aortic arch was not associated with re-CoA in our study (29). This could be explained by the small number of cases in our study.

#### Surgical data

The preferred neonatal repair technique for simple CoA is the end-to-end or extended end-to-end anastomosis via lateral thoracotomy (30), with a lower re-CoA risk for the extended end-to-end anastomosis (31–34). In our cohort, the majority of patients required a more extensive surgical approach via median sternotomy due to either an associated aortic arch hypoplasia and/or VSD in need of neonatal repair. None of these patients developed re-CoA, which is consistent with other studies reporting lower risk for re-CoA in infants undergoing more extensive arch repair with end-to-side or autologous patch material via median sternotomy (35–37). However, other studies found either an increased risk of re-CoA, when patch material was used (5) or no differences in outcome according to the surgical technique used for CoA-repair (6, 27, 38). In a previous nationwide Swedish study including patients operated for CoA between 2011 and 2017, the use of patch material was associated with re-CoA in the univariate analyses. However, after adjusting for other variables associated with re-CoA, this effect was no longer significant (39). Of note, the surgical technique for arch augmentation using patch material was modified at our center in 2018, and the patients who subsequently developed re-CoA all had other types of neonatal CoA repair.

In our study, none of the patients with sternotomy had residual, postoperative hypoplasia of the ascending aorta and transverse aortic arch, which have been associated with an increased risk of re-CoA in previous studies (27, 35). In contrast to earlier studies, there was no association between age <15 days (*n* = 7) nor weight <2.5 kilograms (*n* = 1) at initial CoA-repair and increased risk for re-CoA (4), probably due the small sample size and advances in surgical technique.

#### Postoperative aortic arch geometry, flow anomalies and their relation to re-CoA

##### Postoperative aortic arch geometry including 3d angles

All infants with re-CoA had a crenel arch configuration, which is consistent with previous studies (9, 37). None of the patients with a gothic arch developed re-CoA, which may be attributed to the small number of cases with this arch configuration (*n* = 2) in our study.

Smaller angles between the ascending aorta and the brachiocephalic artery and between the proximal aortic arch and the left common carotid artery were noted in infants with re-CoA. Intuitively, proximal branching vessels more aligned with the distal ascending aorta especially in a more rectangular, crenel-like arch with smaller size distally, seen in most patients with end-to-end or extended end-to-end anastomosis, could result in abnormal flow streaming with consecutively altered flow and re-CoA. However, this could not be demonstrated in our study. A caliber mismatch with larger ascending aorta and brachiocephalic artery and smaller transverse aortic arch may be of greater importance than arch geometry for changes in flow (23) and in the occurrence of re-CoA, as suggested by our findings.

##### Changes in flow pattern in the distal aortic arch following neonatal CoA repair

Our study showed more left-handed systolic helical flow, more right-handed diastolic helical flow, and less vortical flow in infants with re-CoA compared to those who did not develop re-CoA. An MRI study conducted more than 10 years after CoA repair showed no difference in overall helical flow but an increase in local helical and vortical flow in different sections of the aorta compared to healthy controls (10). In contrast to our study, the majority of these patients had undergone resection with end-to-end anastomosis, and none had re-CoA (10). Importantly, these changes were not limited to the specific region of repair (10). In our study, we did not examine the entire aortic arch. Instead, we focused on the distal transverse arch down to the proximal descending aorta including the isthmic region of the aortic arch, where re-CoA usually occurs. Furthermore, our cohort included much younger patients who underwent an MRI scan just days after neonatal CoA-repair and flow patterns were not compared to healthy controls.

The different results we found in our study could be explained by all the above differences in study design between the two studies.

#### Clinical data prior to discharge and their relation to re-CoA

Previous studies have identified higher early, pre-discharge isthmic peak flow velocity and systolic pressure gradient between arm and leg as risk factors for re-CoA (7, 27). In our study, only the peak flow velocity prior to discharge was associated with re-CoA. Some inherent difficulties in reliably measuring blood pressure in infants may be a possible explanation for the lack of differences in blood pressure gradients before discharge.

### Limitations

The main limitation is the low number of patients with re-CoA. Also, no healthy controls were included to examine 3D arch geometry and flow pattern in the native aortic arch.

## Conclusions

MRI-based 3D aortic arch geometry and 4D flow patterns, including helical and vortical flow, can be assessed in neonates after CoA repair and may be a useful method to further investigate the relationship between the complex aortic arch geometry and flow after neonatal CoA repair.

The identified changes in aortic arch geometry and flow pattern after neonatal CoA repair may contribute to the formation of re-CoA. Further studies involving a larger number of patients with re-CoA are needed to evaluate their impact on the development of re-CoA, which may improve outcomes after neonatal CoA repair.

## Data Availability

The raw data supporting the conclusions of this article will be made available by the authors, without undue reservation.
