# Supplementary material for: Three-dimensional aortic arch geometry and blood flow in neonates after surgical repair for aortic coarctation
Source: Front Cardiovasc Med. 2025 Jan 6;11:1518070. doi: 10.3389/fcvm.2024.1518070 (PMC11743609; doi:10.3389/fcvm.2024.1518070)
Supplement: Supplementary file 1 [file Datasheet1.pdf]

SUPPLEMENTARY MATERIAL

Supplementary Table 1: 4D flow imaging sequence parameters.

	**Patient protocol (patients and phantom)**	**High-resolution 2 mm (phantom only)**	**High-resolution 1 mm (phantom only)**
Field of view (read × phase × slice, mm^3)	380 × 285 × 60	320 × 240 × 44	240 × 144 × 44
Acquired spatial resolution (read × phase × slice, mm^3)	2.4 × 3.6 × 1.5	2.0 × 2.0 × 2.0	1.0 × 1.0 × 1.0
Reconstructed spatial resolution (read × phase × slice, mm^3)	2.4 × 2.4 × 1	2.0 × 2.0 × 2.0	1.0 × 1.0 × 1.0
Temporal resolution, acquired (frames per heart beat)	41 ms / 11±1	41 ms / 11±1	43 ms / 10±1
Temporal resolution, reconstructed (frames per heart beat)	20	40	40
TR/TE/flip (ms / ms / °)	5.2 / 2.4 / 7	5.2 / 2.4 / 7	5.4 / 2.6 / 7
VENC, cm/s	Patients: 150<br>Phantom: 200	200	200
Acceleration	GRAPPA R=2	GRAPPA R=2	GRAPPA R=2
Respiratory gating	Yes (in patients)	n/a	n/a

TE = echo time, TR = repetition time, flip = flip angle, VENC = velocity encoding parameter, n/a = not applicable.

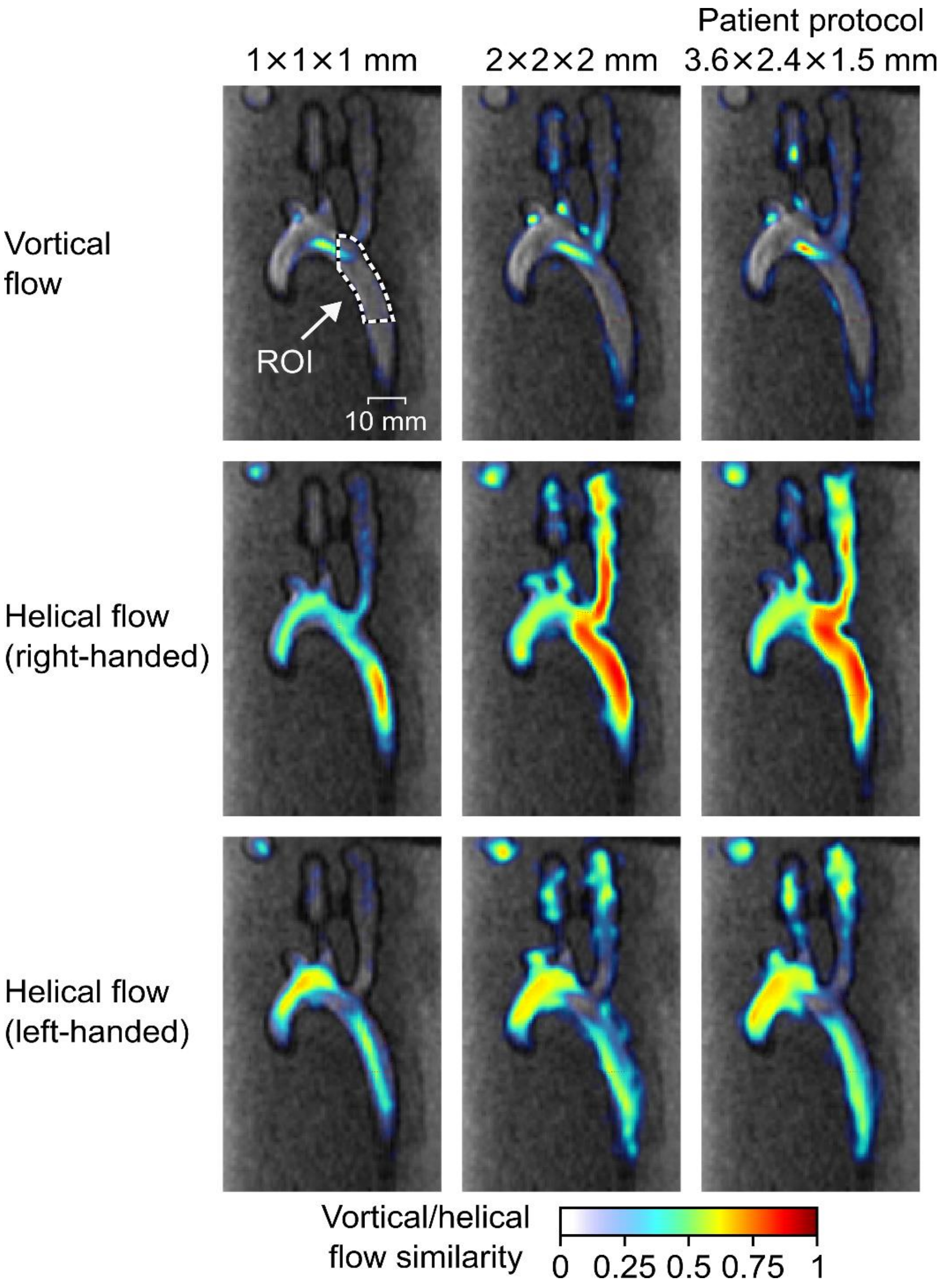

Supplementary Figure 1: Visual comparison of vortical and helical flow for different spatial resolutions in the phantom 4D flow data.

Visually, vortical flow showed comparable results between the three spatial resolutions (1×1×1 mm^3, 2×2×2 mm^3, patient protocol: 3.6×2.4×1.5 mm^3). For left-handed and right-handed helical flow, a lower intensity was found for 1×1×1 mm^3 compared to 2×2×2 mm^3 and the patient protocol. ROI = region of interest where the vorticity and helicity were computed.

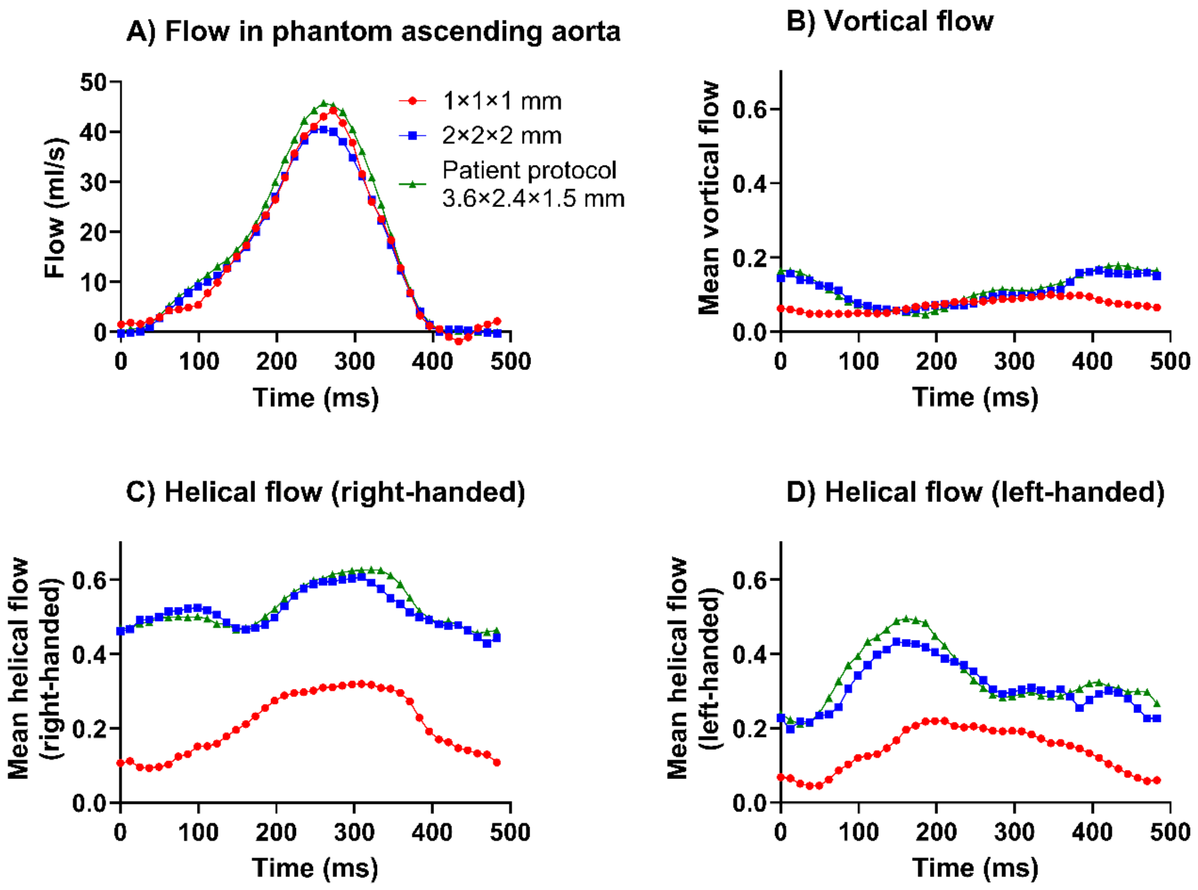

Supplementary Figure 2: Ascending aortic flow rate, and vortical and helical flow in the phantom over the cardiac cycle.

Panel A shows the flow in the ascending aorta in the phantom model for the three different spatial resolutions of the 4D flow (1×1×1 mm^3, 2×2×2 mm^3, patient protocol: 3.6×2.4×1.5 mm^3). Panel B shows vortical flow, Panel C shows right-handed helical flow, and panel D left-handed helical flow. Helical flow was lower for 1×1×1 mm^3 resolution compared to 2×2×2 mm^3 and the patient protocol.
